# Heterogeneous genetic architectures and evolutionary genomics of prostate cancer in Sub-Saharan Africa

**DOI:** 10.21203/rs.3.rs-3378303/v1

**Published:** 2023-10-12

**Authors:** Timothy Rebbeck, Rohini Janivara, Wenlong Chen, Ujani Hazra, Shakuntala Baichoo, Ilir Agalliu, Paidamoyo Kachambwa, Corinne Simonti, Lyda Brown, Saanika Tambe, Michelle Kim, Maxine Harlemon, Mohamed Jalloh, Dillon Muzondiwa, Daphne Naidoo, Olabode Ajayi, Nana Snyper, Lamine Niang, Halimatou Diop, Medina Ndoye, James Mensah, Afua Darkwa-Abrahams, Richard Biritwum, Andrew Adjei, Akindele Adebiyi, Olayiwola Shittu, Olufemi Ogunbiyi, Sikiru Adebayo, Maxwell Nwegbu, Hafees Ajibola, Olabode Oluwole, Mustapha Jamda, Audrey Pentz, Christopher Haiman, Petrus Spies, Andre Van der Merwe, Michael Cook, Stephen J. Chanock, Sonja I. Berndt, Stephen Watya, Alex Lubwama, Mazvita Muchengeti, Sean Doherty, Natalie Smyth, David Lounsbury, Brian Fortier, Thomas Rohan, Judith Jacobson, Alfred Neugut, Ann Hsing, Alexander Gusev, Oseremen Aisuodionoe-Shadrach, Maureen Joffe, Ben Adusei, Serigne Gueye, Pedro Fernandez, Jo McBride, Caroline Andrews, Lindsay Petersen, Joseph Lachance

**Affiliations:** Dana-Farber Cancer Insitute; Georgia Institute of Technology; University of the Witwatersrand; Georgia Institute of Technology; University of Mauritius; Albert Einstein College of Medicine; Centre for Proteomic and Genomic Research; Georgia Institute of Technology; Georgia Institute of Technology; Georgia Institute of Technology; Georgia Institute of Technology; Georgia Institute of Technology; Hôpital Général Idrissa Pouye; Centre for Proteomic and Genomic Research; Centre for Proteomic and Genomic Research; Centre for Proteomic and Genomic Research; 37 Military Hospital; Hôpital Général Idrissa Pouye; Hôpital Aristide Le Dantec; Universite Cheikh Anta Diop de Dakar; University of Ghana Medical School; Korle Bu Teaching Hospital; University of Ghana Medical School; University Of Ghana Medical School; University of Ibadan; University of Ibadan; University of Ibadan; University of Ibadan; University of Abuja Teaching Hospital and Cancer Science Center; University of Abuja Teaching Hospital and Cancer Science Center; University of Abuja and University of Abuja Teaching Hospital; University of Abuja Teaching Hospital and Cancer Science Center; Wits Health Consortium (PTY) Ltd; University of Southern California; Stellenbosch University; Stellenbosch University; National Cancer Institute; National Cancer Institute; Division of Cancer Epidemiology and Genetics, National Cancer Institute, NIH, Bethesda; Mulago Hospital/Makere University; Makerere University College of Health Sciences; National Institute for Communicable Diseases a Division of the National Health Laboratory Service; University of the Witwatersrand; University of the Witwatersrand; Albert Einstein College of Medicine; Dana-Farber Cancer Institute; Albert Einstein College of Medicine; Columbia University; Columbia University; Stanford University School of Medicine; Dana-Farber Cancer Institute; University of Abuja and University of Abuja Teaching Hospital; Wits Health Consortium (PTY) Ltd; 37 Military Hospital; Universite Cheikh Anta Diop de Dakar; Stellenbosch University; Centre for Proteomic and Genomic Research; Dana Farber Cancer Institute; Centre for Proteomic & Genomic Research; Georgia Institute of Technology

**Keywords:** Africa, genetic heterogeneity, health disparities, population genetics, prostate cancer

## Abstract

Men of African descent have the highest prostate cancer (CaP) incidence and mortality rates, yet the genetic basis of CaP in African men has been understudied. We used genomic data from 3,963 CaP cases and 3,509 controls recruited in Ghana, Nigeria, Senegal, South Africa, and Uganda, to infer ancestry-specific genetic architectures and fine-mapped disease associations. Fifteen independent associations at 8q24.21, 6q22.1, and 11q13.3 reached genome-wide significance, including four novel associations. Intriguingly, multiple lead SNPs are private alleles, a pattern arising from recent mutations and the out-of-Africa bottleneck. These African-specific alleles contribute to haplotypes with odds ratios above 2.4. We found that the genetic architecture of CaP differs across Africa, with effect size differences contributing more to this heterogeneity than allele frequency differences. Population genetic analyses reveal that African CaP associations are largely governed by neutral evolution. Collectively, our findings emphasize the utility of conducting genetic studies that use diverse populations.

## Introduction

Prostate cancer (CaP) is the second most commonly diagnosed cancer in men^1^ and highest in men of African descent.^2–4^ In the United States, age-standardized incidence rates of CaP per 100,000 men are 67.3 for Asian Americans, 88.3 for Native Americans, 123.0 for European Americans and 203.2 for African Americans (AA).^5^ CaP mortality rates also vary substantially among global populations of African descent. Age-standardized CaP mortality rates per 100,000 men are 20.2 for West Africa, 16.3 for East Africa, 22.0 for South Africa, and 27.9 for the Caribbean.^1^ KhoeSan ancestry has also been associated with higher CaP risks.^6^

Genetics may explain some of the differences in CaP incidence and mortality rates.^7^ CaP has the highest heritability of all common cancers (51%-63%)^8^ and few modifiable risk factors.^9^ Germline genetic variation is associated with CaP incidence, aggressiveness, and prognosis.^10^ This variation can include private alleles, i.e., population-specific genetic variants. For example, high penetrance founder mutations in *BRCA2* impact CaP risks and responses to treatment in African populations.^11,12^ To date, genome-wide association studies (GWAS) have identified over 450 germline variants that are associated with case/control status.^13–16^ By contrast, there has been less success identifying variants that are associated with aggressive CaP.^17^ Germline variants have also been used to generate polygenic models of CaP risk.^18–20^ However, most genetic studies have focused on individuals of European descent,^21,22^ and existing polygenic risk scores (PRS) for CaP have less satisfactory performance when applied to populations of African descent.^23,24^

It is critical to study disease etiology using data derived from diverse populations, as genetic findings often generalize poorly across populations.^25,26^ Clinical errors (i.e., genetic misdiagnoses) can also occur if findings from one population are applied in a different population.^27^ These issues are compounded by the substantial genetic diversity found in Africa: what is true for one part of the continent may not be true for other parts of Africa.^28^ Population-specific genetic architectures of CaP can arise via locus and allelic heterogeneity.^29^ Effect sizes in Africa can vary by ancestry, either due to genotype-by-environment interactions or epistatic interactions. Prior genetic investigations of CaP in Africa have largely been restricted to studies with limited sample sizes.^6,30–32^ Thus, there is a clear need to study the genetics of CaP at scale in multiple African populations, while also leveraging the emerging genomics capacity within Africa.

To uncover aspects of CaP genetics that are specific to men of African descent, we conducted a pooled analysis of 3,963 CaP cases and 3,509 controls from Senegal, Nigeria, Ghana, Uganda, and South Africa (Fig. S1). This marks the largest African study of its kind, and the majority of the samples studied were genotyped on African soil. On a continental scale we tested for genetic associations with case/control status as well as CaP aggressiveness and inferred functional genomic characteristics of these hits. To identify regional differences in the genetic architecture of CaP, we compared findings from West, East, and South Africa. We then examined whether allele frequency or effect size differences drive regional heterogeneity in the genetic architecture of CaP, before inferring the evolutionary history of African CaP-associated variants.

## Results

### African datasets and population genetics

To maximize statistical power, we combined novel data from the Men of African Descent and Carcinoma of the Prostate (MADCaP) Network with existing data from Uganda and Ghana (Table 1). The MADCaP Network dataset encompasses 2,505 age-matched cases and 2,222 non-cancer controls from urban and suburban locales in Senegal, Nigeria, Ghana, and South Africa.^33^ MADCaP samples were genotyped using a novel genotyping array optimized for fine-mapping and detecting cancer associations in sub-Saharan Africa.^34^ An additional 835 cases and 667 controls from the Uganda Prostate Cancer Study (UGPCS)^30^ were included, as were 623 cases and 620 controls from the Ghana Prostate Cancer Study.^32^ While no exclusion criteria were applied based on disease severity, 35% of cases analyzed that were diagnosed with aggressive forms of CaP (Gleason score ≥ 8). After inferring the optimal imputation panel for each dataset (Fig. S2), a total of 19,858,044 variants were tested for genetic associations with CaP. Additional details about sample accrual, data harmonization, and QC can be found in the Methods and Fig. S1.

**Figure 1:**
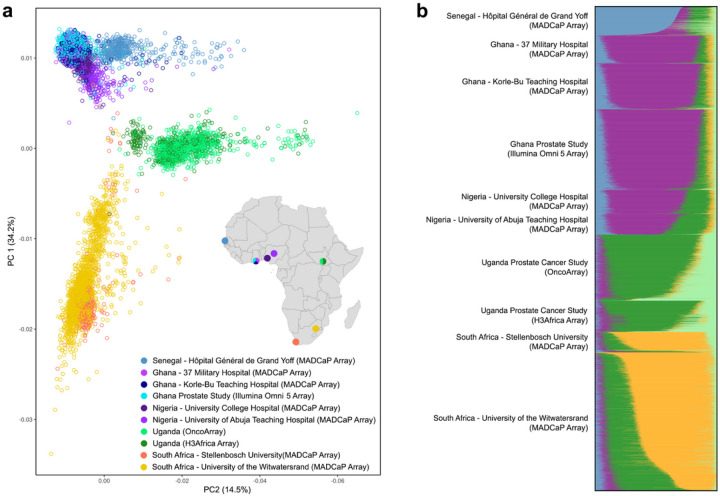
Population genetic structure of African men in this study. **a,** Principal component analysis (PCA) reveals three broad clusters, corresponding to West, East, and South Africa. Colors of points indicate study site and genotype technology. **b,** ADMIXTURE plot showing the shared genetic ancestries of samples (K = 5). Additional values of K are shown in Fig. S3.

The individuals studied here comprise a wide range of ancestries (Table 1). To characterize the genetic diversity found in our pan-African dataset, we visualized samples in principal component analysis (PCA) space (Fig. 1a). Individuals clustered into broad groups according to geography. Although some distinction can be made between samples from Senegal, Ghana, and Nigeria, West African samples clustered together in the upper left part of Fig 1a. Similarly, South African samples clustered together in the lower left part of this figure, while East African samples clustered together in the middle right part of Fig. 1a. Clustering of individuals in PCA space was indifferent to genotype array technology: Ghanaian samples genotyped on the Illumina Omni 5 Array and MADCaP Array clustered together, as did Ugandan samples genotyped using either the H3Africa array or the OncoArray. Shared ancestries across samples were revealed by an ADMIXTURE plot. At K = 5 (Fig. 1b), ancestry components were largely stratified by sampling location: Senegal (blue), Ghana and Nigeria (purple), Uganda (dark and light green), and South Africa (orange). Note that every individual analyzed here contains mixtures of different African ancestries.

### Pan-African GWAS

**Figure 2:**
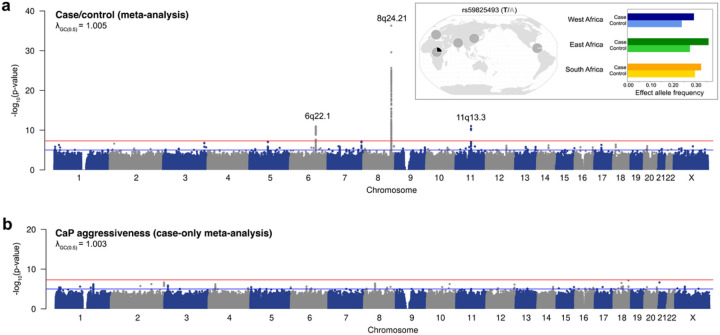
Pan-African GWAS results. Red lines in Manhattan plots mark the genome-wide significant p-value threshold of 5×10^−8^ and blue lines mark the marginally significant p-value threshold of 1×10^−5^. **a,** Case/control pooled meta-analysis of 3,963 African cases and 3,509 African controls. Inset: global and African allele frequencies for rs59825493. **b,** Case-only GWAS of CaP aggressiveness. Sample size: 3,501 African cases with Grade Group information.

A pooled meta-analysis that combined cases and controls from Ghana, Senegal, Nigeria, Uganda, and South Africa yielded 238 genome-wide significant associations with CaP (p-value < 5×10^−8^). After correcting for population structure, study site, and genotype array technology, the genomic inflation factor from this analysis was negligible (1.006, Fig. S4), and there were no significant batch effects. Our pooled meta-analysis yielded 15 independent fine-mapped variants in three loci: 8q24.21, 6q22.1, and 11q13.3 (Fig. 2a and Table 2). Six of these fine-mapped CaP associations featured alleles that are private to Africa, (i.e., they are effectively monomorphic in non-African populations in the 1000 Genomes Project; 1KGP).^35^ For example, rs59825493 at 8q24.21 has a frequency of 0% for the risk-increasing T allele in Europe, East Asia, and South Asia, but a frequency of approximately 26% within Africa. Allele frequencies at rs59825493 vary within the African continent and between cases and controls (Fig. 2b). Four of the fifteen fine-mapped associations (p-value < 5x10^−8^) are not in linkage disequilibrium with any previously known CaP associations: rs114705582, rs7833560, rs61732842, and rs16902043 (Table 2). We also performed a pooled mega-analysis of 3,963 cases and 3,509 controls that incorporated a uniform minor allele frequency filter, which yielded further support for African CaP associations at 8q24.21, 6q22.1, and 11q13.3 (Methods and Fig. S5a). The SNP heritability explained by this pan-African study was 16%.

**Figure 3:**
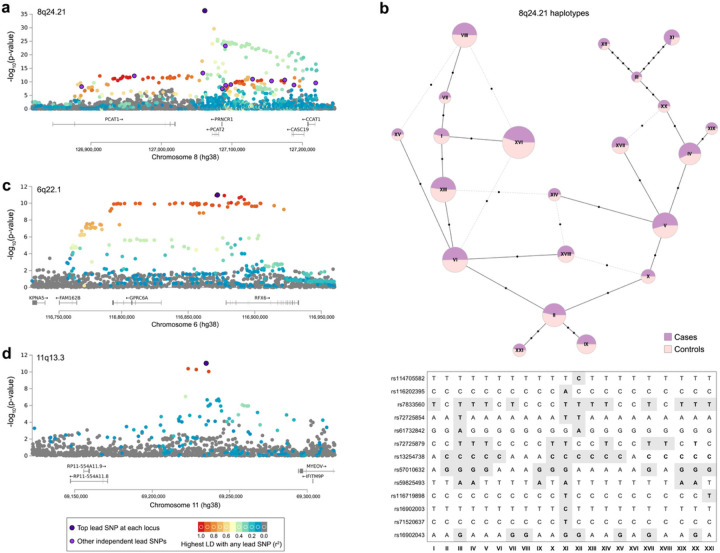
LD patterns for the top three CaP-associated loci. Each point corresponds to a different SNP, with colors indicating the highest LD to any lead SNP at a locus (higher r^2^ values are represented by warmer colors). **a,** 8q24.21 contains thirteen independent fine-mapped SNPs at a p-value threshold of 5×10^−8^. **b,** Haplotype network at 8q24.21. Larger nodes indicate haplotypes that are more common. Proportions of cases and controls are indicated by purple and pink shading, respectively. **c,** 6q22.1 contains one independent fine-mapped SNPs at a p-value threshold of 5×10^−8^. d, 11q13.3 contains one independent fine-mapped SNPs at a p-value threshold of 5×10^−8^.

The 8q24.21 locus contains 214 genome-wide significant variants for case/control status (Fig. 3a). These variants span a 331kb region that contains three recombination hotspots in genomes of African ancestry (chr8:126887955-127219700).^36^ The six SNPs with the strongest p-values in our study (rs72725854, rs59825493, rs116719898, rs16902003, rs71520637, and rs114705582) are Africa-specific polymorphisms. Fine-mapping of the 8q24.21 region revealed thirteen independent CaP-associated SNPs, while conditional analysis using COJO (conditional and joint analysis) implicated rs72725854 and rs72725879. These differences are due to complex patterns of linkage disequilibrium (LD) at 8q24.21, including pairs of SNPs that have low values of r^2^ and high values of D´ (Fig. S6). When we analyzed the lead SNPs at this locus using a haplotype network framework, we found that individuals who inherited haplotype XI (Fig. 3b) had a relatively high risk of CaP (OR = 2.44, 95% CI: 1.90–3.14). Three of the genome-wide significant variants at 8q24.21 are not in LD with known GWAS hits (rs114705582, rs7833560, and rs61732842), while a fourth (rs1948915) is in LD with a SNP that has previously been associated with multiple myeloma.^37^ The nine remaining top hits at 8q24.21 are either exact matches or in LD with previously published CaP GWAS hits. Most notably, the multi-allelic SNP (rs72725854) has been associated with a higher risk of early onset CaP as well as faster disease progression in AA.^38^ Though in a desert of protein-coding genes, the implicated variants at 8q24.21 are close to long non-coding RNA genes that influence cell proliferation, metastasis, and resistance to treatment: *PCAT1*, *PCAT2*, *PRNCR1*, *CASC19,* and *CCAT1*.^39–41^

The 6q22.1 locus contains 60 genome-wide significant associations, including multiple SNPs that are in high LD with prostate eQTLs (Fig. 3c). The credible interval for this region spans chr6:116772177-116920858. Fine-mapping at this locus implicated a single independent SNP that has previously been associated with CaP (rs339321). Twenty of the top 60 SNPs at 6q22.1 are in the intronic region of *RFX6*, a gene that is correlated with tumor progression, metastasis, and biochemical relapse of CaP when upregulated by *HOXB13*.^42^ Similarly, two of the top 60 SNPs at 6q22.1 (rs6901971 and rs2274911) are in the exons of *GPRC6A*, a gene that has been shown to accelerate CaP tumor proliferation.^43^

The 11q13.3 locus contains four genome-wide significant associations for case/control status (Fig. 3d). This locus is proximal to *MYEOV*, a hominid-specific oncogene previously implicated in multiple cancers.^44^ All the associations at 11q13.3 are between *MYEOV* and *RP11-554A11.8*, and fine-mapping of the region reveals one independent SNP (rs11228580), which is a known CaP-associated variant.

### Functional genetics and tests of replication

Relaxing the pooled meta-analysis p-value cutoff to 10^−5^ yielded 604 variants associated with case/control status, of which 90 were independent after LD-pruning (r^2^ < 0.2). This set of 90 LD-pruned hits includes some lead SNPs that reach genome-wide significance (Table S1). SNPs associated with CaP susceptibility in Africa include a mix of novel ancestry-specific associations and variants that replicate previous findings; 23 out of 90 LD-pruned associations are in linkage disequilibrium with previously reported CaP hits (r^2^ < 0.2, Table S1). Noteworthy marginal associations include intronic variants in genes related to male infertility (rs4323394 in *GALNTL5*),^45^ breast cancer (rs116541708 in *ATP2B2*),^46^ and prostate cancer cell invasion (rs142311960 in *ECE1*).^47^ Reactome^48^ pathways showing the greatest enrichment for CaP-associated variants included collagen chain trimerization, vitamin C metabolism, amine ligand-binding receptors, and reduction of cytosolic Ca^2+^ levels (Table S2). Many African CaP-associated loci colocalize with ChIP-seq peaks from DNA binding experiments for transcription factors. Five out of 90 independent CaP associations overlapped with HOXB13 binding regions, 12 overlapped with MYC binding regions, and 11 overlapped with AR binding regions (Table S3).

We also conducted both a Regulome-Wide Association (RWAS) and a Transcriptome-Wide Study (TWAS) to identify regulatory elements that are genetically correlated with CaP risk. RWAS analysis yielded summary statistics for 54,410 features that overlapped variants in our study. Two of these features reached genome-wide significance (Fig. S7a). The first feature is associated with the regulation of prostate adenocarcinoma: PRAD_53245 at 8q24.21 (RWAS p-value = 5.76×10^−14^). The second feature is associated with regulation of pheochromocytoma and paraganglioma cancer: PCPG_30379 at 6q22.1 (RWAS p-value = 1.81×10^−11^). Our TWAS identified two genome-wide significant SNPs: rs339321 and rs2274911 (TWAS p-values = 5.17×10^−9^ and 6.28×10^−9^, Fig. S7b). These TWAS hits are located at 6q22.1 and associated with the expression of *ZUFSP* in the kidney cortex and *FAM162B* in lung squamous cell carcinoma. We note that some SNPs that are significantly associated with regulatory activities need not be significantly associated with gene expression, a phenomenon that is at least partially due to the underrepresentation of African ancestry samples in GTEx. The lack of Africa-specific eQTLs in GTEx, especially at 8q24.21, also likely contributes to why we did not observe any genome-wide significant prostate TWAS hits (Fig. S7c). Nevertheless, there is an overlap between RWAS and TWAS hits generated from different continental datasets. Specifically, European RWAS and TWAS associations with elevated test statistics in Europe were enriched for elevated test statistics in Africa (Fig. S8). Although RWAS and TWAS replication analyses do not consider the direction of effect, this trans-ancestry enrichment is indicative of shared genetic effects between Europe and Africa at the same functional variants.

**Figure 4:**
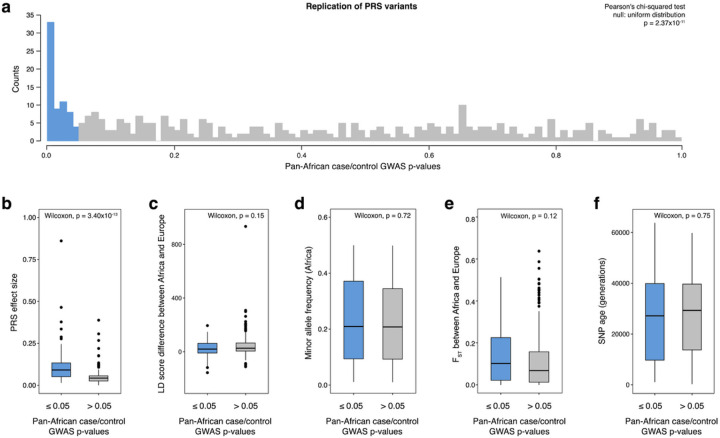
Replication of PRS variants in sub-Saharan Africa. Here, we focus on how well PRS variants from Wang et al.^49^ replicate in our pan-African meta-analysis of CaP cases and controls. **a,** Previously identified PRS variants are enriched for low p-values in our case/control GWAS (under a null hypothesis of no replication p-values would be uniformly distributed). Bins are colored by pan-African case/control p-value (≤ 0.05 in blue and > 0.05 in gray). Panels **b-f** examine whether different features of PRS variants from Wang et al.^49^ PRS are associated with lower p-values in our study (Wilcoxon rank-sum tests): b, Effect sizes (PRS weights); **c,** LD score differences between Europe and Africa (LD score_EUR_ – LD score_AFR_); **d,** Minor allele frequencies in Africa; **e,** F_ST_ between Europe and Africa (higher values are indicate of larger allele frequency differences between EUR and AFR in the 1KGP); **f,** SNP age, in generations.

Using results from our pooled meta-analysis of cases and controls, we tested how well genetic variants from a leading PRS replicated in Africa. The PRS for CaP by Wang et al.^49^ contains 451 CaP-associated variants that were ascertained in a multi-ethnic cohort (12.4% of the cases and 7.8% of the controls used to generate this PRS were of African descent). Under a null hypothesis of no-replication, PRS variants are expected to have p-values that are uniformly distributed. However, we observed substantial enrichment for low p-values in our pooled meta-analysis of African cases and controls (Fig. 4a, p-value < 2.37×10^−11^). Genetic variants from the Wang et al.^49^ PRS were 6.05 times more likely to have a p-value < 0.05 than expected by chance. Further examining the characteristics of CaP associations that replicate across continental ancestries, we found that variants with large PRS weights (i.e., effect sizes) were enriched for low p-values in our pooled meta-analysis of African cases and controls (Fig. 4b). However, LD score differences, minor allele frequencies, F_ST_ between Africa and Europe, and allele age did not have a large effect on whether PRS variants were associated with CaP in sub-Saharan Africa (Fig. 4c–f).

### CaP aggressiveness

We performed a case-only meta-analysis of CaP aggressiveness using Grade Group^50^ as a classifier of CaP aggressiveness. CaP was classified as non-aggressive if cases were in Grade Group 1 (Gleason score ≤ 6, n = 712), mildly aggressive if cases were in Grade Groups 2 or 3 (Gleason score = 7, n = 1390), and severely aggressive if cases were in Grade Groups 4 or 5 (Gleason score ≥ 8, n = 1399). Although no associations with CaP aggressiveness reached genome-wide significance, several peaks of marginal significance (p-value < 10^−5^) were found (Fig. 2b). These marginally significant hits included rs149639001 and rs78479840, which are adjacent to pseudogenes. A third marginally significant aggressiveness hit, rs190761537, is in the intronic region of the *SNTG1*, a gene that is involved in cell communication. An additional GWAS contrasting severe cases (Grade Groups 4 to 5) with controls yielded no additional genome-wide significant associations apart from the ones already implicated in the main case/control analysis (Fig. S5b). Finally, we note that the marginal peaks associated with CaP aggressiveness are in different genomic regions than the peaks associated with case/control status (Fig. 2). This suggests that CaP aggressiveness has a different genetic architecture than case/control status. Subsequent analyses focused on variants that were associated with case/control status.

### Regional differences across Africa

To compare the genetic architectures of CaP across Africa, we juxtaposed case/control GWAS results from West (1,780 cases; 1,739 controls), East (835 cases; 667 controls), and South Africa (1,348 cases; 1,103 controls). A strong GWAS peak was observed at 8q24.21 for all three regional GWAS (Fig. S5c-e). However, the continent-wide peak at 6q22.1 is largely driven by a West African signal, and the continent-wide peak at 11q13.3 is due to a combination of marginal associations in each region. The West African GWAS also yielded an additional peak at the 6q21 locus (regional p-value = 3.35×10^−8^), with variants in the intronic region of *MTRES1* which is a mitochondrial transcription regulator (lead SNPs: rs77426886 and rs147055316). Comparisons of Manhattan plots for West (Fig. S5c), East (Fig. S5d), and South Africa (Fig. S5e) reveal differences in the genomic loci that are marginally significant in each region. This suggests that locus heterogeneity may contribute to differences in the genetic architecture of CaP across Africa. Notable marginally significant (p-value < 1×10^−5^) region-specific associations include rs150430268 at the *RGS6* gene (an essential tumor suppressor)^51^ in West Africa, rs144499050 in the gene *PTPN2* (a known regulator of inflammation and cancer)^52^ in East Africa, and rs7905960 at the *ADAM12* gene (a predictor of chemoresistance and metastasis in ER-negative breast cancer)^53^ in South Africa.

### Heterogeneity in CaP genetic architecture across Africa

**Figure 5:**
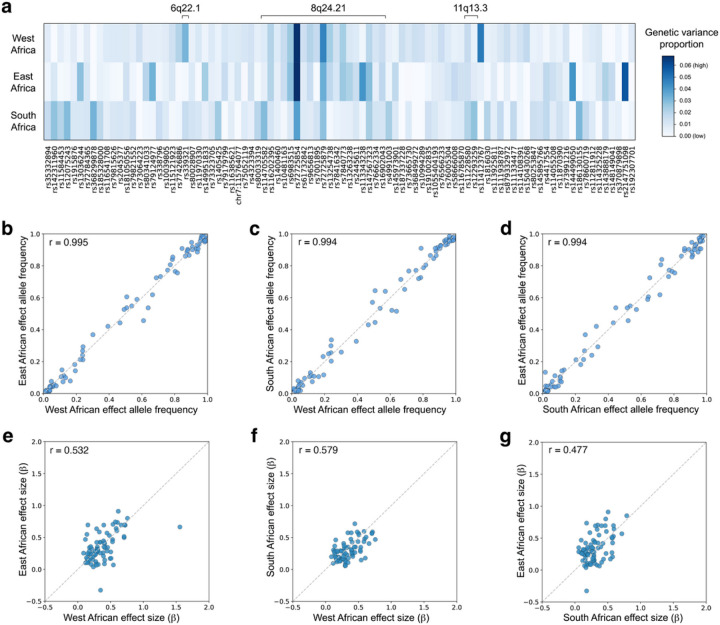
Regional heterogeneity in the genetic architecture of CaP. These plots focus on 90 independent marginally associated variants (p-value threshold: 1×10^−5^). Note that LD pruning (r^2^ < 0.2) yields a slightly different set of variants at 8q24.21 than fine-mapping. **a,** Heatmap quantifying the relative contributions of 90 CaP-associated variants to the genetic variance of case/control status (*gvp*, see Methods). **b,** Allele frequencies of CaP-associated variants in West and East Africa. **b,** Allele frequencies of CaP-associated variants in West and South Africa. **c,** Allele frequencies of CaP-associated variants in East and South Africa. **d,** Effect size comparisons between West and East Africa. **e,** Effect size comparisons between West and South Africa **f,** Effect size comparisons between East and South Africa. Scatterplots of effect sizes require that variants are polymorphic in both populations.

To further explore differences in the genetic architecture of CaP across West, East, and South Africa, we compared the relative importance of different genomic loci by calculating the contribution of the top 90 independent associations (p-value < 1×10^−5^) to the genetic variance of CaP. For each region, this yielded a set of 90 genetic variance proportions (*gvp*). Visualizing *gvp* statistics in a heatmap reveals both similarities and differences in the genetic architecture of CaP across Africa (Fig. 5a). While the 8q24.21 locus is a key driver of CaP risk in all three regions of Africa, its relative importance is larger in West and East Africa than in South Africa. Other marginal CaP associations exhibit population-specificity in their *gvp*, including X-linked variants that contribute more to CaP risk in East Africa than in other regions (Table S1). To formally test whether the genetic architecture of CaP differs across Africa, we incorporated uncertainty in allele frequencies and effect sizes into our *gvp* statistics. Using this framework, the genetic architectures of CaP in West, East, and South Africa were represented as three clouds of points in a multidimensional space (Fig. S9). Notably, there was no overlap between any of the clouds of points, i.e., each region of Africa has a distinct genetic architecture (ANOSIM F = 0.8865, p-value = 0.001).

We further explored regional differences in the genetic architectures of CaP by comparing allele frequency and effect size estimates across Africa. Pairwise comparisons reveal that allele frequencies of CaP-associated variants are broadly similar in West, East, and South Africa (Fig. 5b–d). By contrast, substantial differences in effect sizes are observed across the continent (Fig. 5e–g). However, effect size estimates tend to be much noisier than allele frequency estimates. Incorporating noise in these estimates, we isolated the impact of effect sizes and allele frequencies on the distances between regional genetic architectures (Fig. S10). This sensitivity analysis confirmed that differences in effect sizes, rather than allele frequencies, drive differences in the genetic architecture of CaP across Africa (t-test p-values < 2×10^−36^ for all pairwise comparisons). Genetic variants exhibiting substantial heterogeneity across regional sub-studies, i.e., large *I*^2^ statistics, include rs339321 and rs72725834 (Table S1).

### Population and evolutionary genetics of CaP

**Figure 6:**
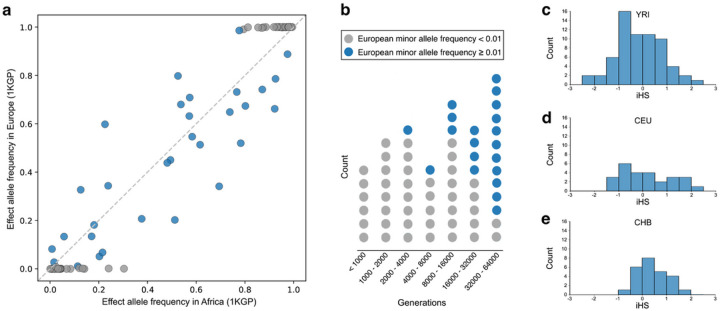
Population and evolutionary genetics of CaP-associated variants. These plots focus on independent marginally associated variants (p-value threshold: 1×10^−5^) that overlap with 1KGP SNPS. **a,** Allele frequencies of CaP-associated variants in Europe and Africa (1KGP data, 87 SNPs). Gray circles indicate variants that are not polymorphic in Europe (minor allele frequency < 0.01). **b,** Ages of 63 CaP-associated variants estimated using GEVA in African populations from the 1KGP data. Gray circles indicate SNPs that are not polymorphic in Europe. **c-e,** Tests of recent natural selection acting on CaP-associated variants in Africa, Europe, and East Asia (**c,** YRI: Yoruba in Ibadan, Nigeria; **d,** CEU: Utah residents with Northern and Western European Ancestry; **e,** CHB: Han Chinese in Beijing). Because iHS statistics require minor allele frequencies > 1% in the target population, our analysis was restricted to 65 CaP-associated variants in Africa, 26 variants in and 25 variants in East Asia. iHS scores quantify signatures of natural selection, and they follow a standard normal distribution under a null hypothesis of neutral evolution. CaP-associated variants are not enriched for extreme iHS scores (i.e., z-scores ≤ −2 or ≥ 2).

Evolutionary history informs our understanding of the genetic architecture of CaP. Focusing on independent marginal associations (p-value < 1×10^−5^), we compared allele frequencies in Europe and Africa. CaP-associated variants ascertained in our pan-African GWAS include polymorphisms that have intermediate allele frequencies across the globe as well as polymorphisms where non-African populations are near monomorphic for either the protective or risk-increasing allele (Fig. 6a). Using GEVA,^54^ we found that allele ages of CaP associated variants in Africa have a wide range (Fig 6b), with the youngest having a median age of ~275 generations (rs116785870) and the oldest with a median age of ~59,000 generations in African populations (rs1405425). Allele age and frequency information can be combined to explain why many CaP-associated alleles are private to Africa. Some CaP-associated variants are due to recent mutations that have not had enough time to diffuse into Europe and other continents (Fig. 6b). Other CaP-associated variants predate the out-of-Africa migration while having negligible allele frequencies in Europe (gray circles with allele ages > 4,000 generations in Fig. 6b). Population bottlenecks and founder effects likely contribute to continental differences in the distribution of these CaP-associated alleles.

We also examined whether natural selection contributes to the heterogeneous genetic architectures of CaP. On a continental scale, this involved testing for recent positive selection via integrated haplotype scores (iHS).^55^ Normalized iHS values are z-statistics, and SNPs are hypothesized to follow a standard normal distribution if they are not governed by selection. For the 1KGP populations analyzed here (Fig. 6c–e) we were unable to reject the null hypothesis of neutral evolution for the set of African-ascertained CaP variants (Shapiro-Wilk test of normality, p-values = 0.893, 0.555, and 0.893 for YRI, CEU, and CHB, respectively). Given this absence of strong selection, the out-of-Africa bottleneck may be one key reason why private African alleles are observed. Within Africa, we calculated population-branch statistics (PBS) for each CaP-associated variant. These statistics identify SNPs with particularly large allele frequency differences across African populations. However, none of the top 90 CaP-associated variants were outliers when compared to genome-wide distributions of PBS statistics (Table S1). Thus, population-level heterogeneity in the genetic architecture of CaP appears to be largely due to genetic drift, as opposed to natural selection. This is consistent with the late age of CaP onset (i.e., past typical reproductive ages) and previous studies that focused on European-ascertained CaP variants.^7,23^

## Discussion

Our findings underscore the critical importance of conducting genetic studies of disease etiology in diverse populations. By examining the genetics of CaP across Africa, we identified novel associations that could not have been detected in a non-African GWAS. Although the top hits implicated in our study overlap genomic regions that contain known cancer loci (8q24.21, 6q22.1, and 11q13.3), we find evidence of substantial allelic heterogeneity, as many of the lead variants associated with CaP in Africa differ from those found in other continents. Furthermore, the relative importance of each independent CaP association varies across African populations. These differences in genetic architecture are due to multiple evolutionary phenomena, including recent mutations, genetic drift, and population bottlenecks. Our data also revealed novel CaP-associated haplotypes with large effect sizes that are unique to African populations. The existence of private alleles and population-specific effect sizes therefore necessitate studies that include individuals from a broad range of ancestries.

Until recently there has been limited evidence of germline variants that predispose to aggressive prostate cancer. Few SNPs from GWAS have been associated with higher grade or stage disease. However, men of African ancestry in the top decile of a multi-ancestry PRS including 278 risk variants had a significantly higher risk of aggressive CaP (OR = 1.23).^56^ We similarly did not identify significant single variant associations with aggressive disease. This observation is important because our cases had not undergone screening for prostate cancer and were mostly Grade Group 2 or higher. This suggests that prior studies of aggressive CaP did not fail to detect associations because of the nature of their sampling design. Additionally, we note that rs62113212, an intronic variant in *KLK3* that has previously been associated with aggressive CaP,^57^ is near monomorphic in Africa - which explains why it did not come up in our GWAS of different grade groups.

Our results also have implications for the genetic architecture of CaP (and possibly other diseases) in AA. Due to the historical legacy of the trans-Atlantic slave trade, AA are genetically most similar to the West African subset of our data. However, one key difference between African and AA populations is that the latter contain admixed genomes with European and Native American ancestry.^58^ AA also experience substantively different lifestyles and environmental exposures than individuals living in sub-Saharan Africa. These differences may explain why some of our fine-mapped GWAS hits were not implicated in prior studies. While this may in part be due to limited power to detect small effect sizes, other explanations are possible, including epistatic interactions with different genetic backgrounds and genotype-by-environment interactions. Rare variants are also thought to play a large role in the genetic etiology of CaP.^59^ Because these variants tend to have a recent evolutionary origin and limited geographic breadth, future studies focusing on diverse African populations are likely to benefit from increased sample sizes and/or family-based approaches.

The results presented here advance our understanding of the genetic etiology of a complex disease which has an uneven burden across the globe and within the African continent. It is important to study disease associations in populations that have the highest disease burden, as they may harbor alleles that are missing from other, lower-risk, populations. Because CaP screening is essentially non-existent in Africa, the natural history of CaP (and genetic associations of CaP) can be studied in the absence of early detection. The presence of private alleles helps explain why existing polygenic scores for CaP are less portable to men of African descent. Evolutionary analyses explain why some CaP variants are not seen in European populations, and thus in part explain the higher incidence of CaP in unscreened high-risk African populations. Our study demonstrates the benefits of conducting genetic studies in diverse understudied populations. Future studies will benefit from polygenic predictions that utilize ancestry-specific information, helping to remedy existing disparities in genomic medicine.

## Supplementary Material

Supplement 1

## Figures and Tables

**Table 1.** Selected characteristics of CaP cases and controls.

Participant characteristics	Cases (n = 3963)	Controls (n = 3509)
Recruitment Center (genotyping platform)		
Senegal: Hôpital Général de Grand Yoff (MADCaP Array)	223 (5.6%)	228 (6.5%)
Ghana: 37 Military Hospital (MADCaP Array)	210 (5.3%)	217 (6.2%)
Ghana: Korle-Bu Teaching Hospital (MADCaP Array)	372 (9.4%)	336 (9.6%)
Ghana Prostate Study (Illumina Omni 5 Array)	623 (15.7%)	620 (17.7%)
Nigeria: University College Hospital (MADCaP Array)	190 (4.8%)	177 (5.04%)
Nigeria: University of Abuja Teaching Hospital (MADCaP Array)	162 (4.1%)	161 (4.6%)
Uganda Prostate Cancer Study (OncoArray)	548 (13.8%)	471 (13.4%)
Uganda Prostate Cancer Study (H3Africa Array)	287 (7.2%)	196 (5.6%)
South Africa: Stellenbosch University (MADCaP Array)	183 (4.6%)	132 (3.8%)
South Africa: University of the Witwatersrand (MADCaP Array)	1165 (29.4%)	971 (27.7%)
Age in years (at recruitment)		
<60	567 (14.3%)	1074 (30.6%)
60–69	1483 (37.4%)	1479 (42.2%)
70–79	1491 (37.6%)	759 (21.6%)
≥80	410 (10.4%)	170 (4.8%)
Data Unavailable	12 (0.3%)	27 (0.8%)
Grade Group		
1	712 (18.0%)	n/a
2+3	1390 (35.1%)	n/a
4+5	1394 (35.2%)	n/a
Data unavailable	467 (11.8%)	n/a
Family history		
First-degree relative	225 (5.7%)	80 (2.3%)
None	2001 (50.5%)	2047 (58.3%)
Data unavailable	1737 (43.8%)	1382 (39.4%)

**Table 2.** Lead GWAS SNPs associated with CaP in African men

SNP ID	Position (hg38)	Effect Allele	Odds Ratio	P-value	West freq.	East freq.	South freq.	EUR freq.
rs72725854	chr8:127062570	T	1.96 (1.76–2.17)	5.08×10^−37^	0.068	0.062	0.081	0.00
rs72725879	chr8:127091724	C	1.42 (1.32–1.52)	5.21×10^−24^	0.657	0.626	0.703	0.802
rs7833560	chr8:127059774	T	1.28 (1.20–1.37)	5.70×10^−14^	0.481	0.491	0.425	0.773
rs116202395	chr8:126962668	C	1.82 (1.55–2.14)	6.14×10^−13^	0.964	0.969	0.987	1.000
rs11228580	chr11:69234875	C	1.32 (1.22–1.44)	9.26×10^−12^	0.169	0.157	0.207	0.176
rs59825493	chr8:127130257	T	1.28 (1.19–1.37)	1.01×10^−11^	0.762	0.723	0.700	1.000
rs339321	chr6:116871027	G	1.32 (1.22–1.43)	1.07×10^−11^	0.779	0.755	0.767	0.729
rs16902003	chr8:127175810	C	1.43 (1.29–1.59)	2.03×10^−11^	0.076	0.115	0.085	0.00
rs116719898	chr8:127157082	C	1.48 (1.32–1.67)	4.63×10^−11^	0.934	0.894	0.948	0.999
rs16902043	chr8:127219700	G	1.26 (1.17–1.36)	2.49×10^−10^	0.215	0.260	0.314	0.341
rs57010632	chr8:127099472	G	1.23 (1.15–1.31)	1.08×10^−9^	0.515	0.511	0.391	0.036
rs71520637	chr8:127188945	C	1.38 (1.24–1.53)	1.44×10^−9^	0.895	0.924	0.923	0.999
rs13254738	chr8:127092098	A	1.24 (1.16–1.33)	1.82×10^−9^	0.347	0.306	0.355	0.665
rs114705582	chr8:126887955	C	1.73 (1.44–2.09)	5.85×10^−9^	0.027	0.012	0.02	0.000
rs61732842	chr8:127087290	G	1.41 (1.25–1.59)	3.09×10^−8^	0.934	0.962	0.917	1.000

Fifteen independent fine-mapped SNPs reached genome-wide significance in our pan-African meta-analysis of CaP cases and controls. Odds ratios include 95% confidence intervals and allele frequencies indicate the frequency of effect alleles in controls from West Africa, East Africa, South Africa, and Europe.
