# Supplementary material for: Heterogeneous genetic architectures and evolutionary genomics of prostate cancer in Sub-Saharan Africa
Source: Res Sq. 2023 Oct 12:rs.3.rs-3378303. Preprint. [Version 1] doi: 10.21203/rs.3.rs-3378303/v1 (PMC10602179; doi:10.21203/rs.3.rs-3378303/v1)
Supplement: Supplement 1 [file NIHPPrs3378303v1-supplement-1.pdf]

Supplementary Files

This is a list of supplementary files associated with this preprint. Click to download.

- MADCaPSupplementalMaterialNatGenv1.docx
